# COVID-19 in multiple-myeloma patients: cellular and humoral immunity against SARS-CoV-2 in a short- and long-term view

**DOI:** 10.1007/s00109-021-02114-x

**Published:** 2021-10-18

**Authors:** Ivana von Metzler, Julia Campe, Sabine Huenecke, Marc S. Raab, Hartmut Goldschmidt, Ralf Schubert, Holger F. Rabenau, Sandra Ciesek, Hubert Serve, Evelyn Ullrich

**Affiliations:** 1 Department of Hematology and Oncology, Johann Wolfgang Goethe University, Frankfurt, Germany; 2 Experimental Immunology, Department for Children and Adolescents Medicine, University Hospital Frankfurt, Goethe University, Frankfurt am Main, Germany; 3 Division of Pediatric Stem Cell Transplantation and Immunology, Department for Children and Adolescents Medicine, University Hospital Frankfurt, Goethe University, Frankfurt am Main, Germany; 4 Department of Medicine V, University Hospital Heidelberg, and National Center for Tumor Diseases (NCT), Heidelberg, Germany; 5 Division for Allergy, Pneumology and Cystic Fibrosis, Department for Children and Adolescents, University Hospital, Goethe-University, Frankfurt, Germany; 6 Institute for Medical Virology, University Hospital Frankfurt, Goethe University, Frankfurt am Main, Germany; 7 German Centre for Infection Research, External Partner Site, 60323 Frankfurt, Germany; 8 Fraunhofer Institute for Molecular Biology and Applied Ecology (IME), Branch Translational Medicine and Pharmacology, 60596 Frankfurt, Germany; 9 German Cancer Consortium (DKTK) partner site Frankfurt/Mainz, Frankfurt am Main, Germany; 10 Frankfurt Cancer Institute (FCI), Goethe University, Frankfurt am Main, Germany

**Keywords:** Multiple myeloma, SARS-CoV-2, COVID-19, Immune response

## Abstract

**Abstract:**

Multiple myeloma patients are often treated with immunomodulatory drugs, proteasome inhibitors, or monoclonal antibodies until disease progression. Continuous therapy in combination with the underlying disease frequently results in severe humoral and cellular immunodeficiency, which often manifests in recurrent infections. Here, we report on the clinical management and immunological data of three multiple-myeloma patients diagnosed with COVID-19. Despite severe hypogammaglobulinemia, deteriorated T cell counts, and neutropenia, the patients were able to combat COVID-19 by balanced response of innate immunity, strong CD8+ and CD4+ T cell activation and differentiation, development of specific T-cell memory subsets, and development of anti-SARS-CoV-2 type IgM and IgG antibodies with virus-neutralizing capacities. Even 12 months after re-introduction of lenalidomide maintenance therapy, antibody levels and virus-neutralizing antibody titers remained detectable, indicating persisting immunity against SARS-CoV-2. We conclude that in MM patients who tested positive for SARS-CoV-2 and were receiving active MM treatment, immune response assessment could be a useful tool to help guide decision-making regarding the continuation of anti-tumor therapy and supportive therapy.

**Key messages:**

- Immunosuppression due to multiple myeloma might not be the crucial factor that is affecting the course of COVID-19.
- In this case, despite pre-existing severe deficits in CD4+ T-cell counts and IgA und IgM deficiency, we noticed a robust humoral and cellular immune response against SARS-CoV-2.
- Evaluation of immune response and antibody titers in MM patients that were tested positive for SARS-CoV-2 and are on active MM treatment should be performed on a larger scale; the findings might affect further treatment recommendations for COVID-19, MM treatment re-introduction, and isolation measures.

**Supplementary information:**

The online version contains supplementary material available at 10.1007/s00109-021-02114-x.

## Introduction

Secondary immunodeficiency is a common feature in multiple myeloma (MM) patients. Hypo-gammaglobulinemia, neutropenia, reduced T and NK cell counts, and impaired T and NK cell function are disease- and/or therapy-induced factors that can contribute to the acquisition of severe bacterial and viral infections with adverse outcomes. From that point of view, we presumed that the SARS-CoV-2 pandemic [1] would place these patients at high risk for unfavorable outcome. Indeed, the first US study on 100 MM patients from NYC reported mortality rates of almost 20% of patients, which was considerably higher than what had been reported in general population [2]. In contrast, the German multiple myeloma study group consortium reported no casualties among all 21 myeloma patients diagnosed with COVID-19 from March 1 to May 31, 2020 at secondary and tertiary comprehensive cancer centers in Germany [3]. So far, no myeloma-specific risk factors have been identified [2, 3]. Despite the lack of reliable data, few new guidelines and recommendations for treatment of MM during COVID-19 pandemic have been published [4]. Here, we report on the clinical management and immunological data over a 12-month period of our first multiple myeloma patient diagnosed with COVID-19.

## Methods

Patient’s medical records and standard laboratory parameters including immune monitoring and chest CT imaging were collected and analyzed for this study. Written patient’s consent was obtained for publication of this brief report. For further non-standard analyses, blood sera were obtained from the here described patient and from two more MM patients after COVID-19 diagnosis and, in one case, lenalidomide-based treatment. In addition, we collected blood sera from an otherwise healthy positive control patient suffering from severe COVID-19, from another control patient with moderate COVID-19 symptoms, and from an age-matched convalescent control. Blood sample collection was in accordance with the Declaration of Helsinki and approved by the Ethics Committee of the University Hospital Frankfurt. All subjects provided written, informed consent.

### Cytokine-bead-array for measurement of serum cytokine concentrations

For cytokine analysis, patients’ sera were collected at the respective days and frozen at −80 °C. Cytokine concentrations were examined using BD cytometric bead array (CBA; BD Bioscience). The tests were performed according to the manufacturer’s instructions. Three hundred events were analyzed per condition. Data were acquired with the BD FACSVerse Bioanalyzer and were quantitated using the FCAP Array software (v3.0.1; BD Biosciences).

### IFN-γ ELISpot assay

Peripheral blood mononuclear cells (PBMCs) were thawed 1 day before seeding to the ELISpot plate and rested overnight. The IFN-γ ELISpot (Mabtech, Nacka Strand, Sweden) was performed in filterplates (MSIPS4510, Merck Millipore, Burlington, USA) according to the manufacturer’s instructions. PBMCs were seeded with a concentration of 3 × 10^5^ cells/100 µL/well in X-Vivo 10 medium supplemented with 2% human AB serum and co-cultured with the following stimuli for 24 h: 25 ng/mL purified anti-CD3 (clone OKT-3; positive control) and a mix of 1.25 µg/mL S-Protein, M-Protein, and N-Protein Peptivator (Miltenyi, Bergisch-Gladbach, Germany; SARS-CoV-2 specific response)), respectively, of medium alone (negative control). Measurements were performed in triplicates. Quantification of spot forming units (SFU)/3 × 10^5^ PBMCs was performed with the ELI-Analyze ELISpot Image Analysis Software from A.EL.VIS. and normalized to the unspecific response (SFU/3 × 10^5^ PBMCs without stimulus).

### Qualitative and quantitative SARS-CoV-2 IgG/IgM measurement

Serum samples from the patient were collected at days + 11, 21, 29, 44, 56, 82, 148, 174, 278, and 356 after COVID-19 diagnosis, respectively.

For qualitative detection of SARS-CoV-2 nucleocapsid (N)-protein–specific IgG (SARS-CoV-2-IgG, chemiluminescent microparticle immunoassay (CMIA), Abbott), SARS-CoV-2 Spike (S) protein–specific IgM (SARS-CoV-2-IgM, Abbott) antibodies and for quantitative detection of SARS-CoV-2 Spike IgG (Abbott SARS-CoV-2 IgG II Quant CMIA), the automated Abbott Alinity i platform (Abbott GmbH, Wiesbaden, Germany) was used according to the manufacturer’s recommendation.

The latter assay measures antibody targeted against the SARS-CoV-2 S protein receptor-binding domain (RBD). Test results are expressed as standardized binding antibody units (BAU)/mL, calibrated to the WHO International Standard for anti-SARS-COV-2 immunoglobulin (human) (NIBSC Code 20–136). The manufacturer’s cut-off for positivity is set to 7.1 BAU/mL. To exclude a false-positive result, a qualitative verification assay (Vircell COVID‐19 ELISA IgG; Vircell Spain S.L.U., Granada, Spain) was used. The assay uses SARS-CoV-2 recombinant S and additionally N protein.

### PRNT for quantification of neutralizing anti-SARS-CoV-2 antibodies

To test for the neutralizing capacity of SARS-CoV-2-specific antibodies, Caco-2 cells (human colon carcinoma cells, ATCC DSMZ ACC-169 (American Type Culture Collection, Manassas, Virginia, USA)) were seeded on a 96-well plate 3–5 days prior to infection. Twofold dilutions of the test sera beginning with a 1:10 dilution (1:10; 1:20; 1:40; 1:80; 1:160; 1:320; 1:640, and 1:1280) were made in culture medium (minimum essential medium, MEM; Sigma-Aldrich, St. Louis, USA) before mixed 1:1 with 100 TCID50 (Tissue culture infectious dosis 50) of reference virus (SARS-CoV-2 FFM1 isolate). FFM1 was isolated from a patient at University Hospital Frankfurt who was tested positive for SARS-CoV-2 by PCR. The test was performed as described earlier [5, 6].

## Results and discussion

The patient, a 51-year-old male, presented with a 3-day fever of 38.8 °C, dry cough, and chills on March 25, 2020. Other symptoms frequently reported in patients with COVID-19 were denied [7]. In January 2019, he was diagnosed with multiple myeloma (MM) type IgA kappa, R-ISS stage I, and 1/4-CRAB criteria. Initially, he was treated with an induction quadruplet consisting of an anti-CD38 antibody in combination with bortezomib, lenalidomide, and dexamethasone, followed by high-dose chemotherapy (HDCT) with melphalan 200 mg/m^2^ and autologous stem cell transplant (ASCT) in September 2019. He achieved complete remission after ASCT but remained MRD positive by flow cytometry (sensitivity 10^−5^). From January 2020 onward, he received continuous lenalidomide maintenance treatment.

During monthly follow-up examinations from the beginning of the maintenance treatment, we noticed severe type IgA and IgM immunoparesis, CTC grade II neutropenia, and CTC grade II lymphocytopenia. In January 2020, we also noticed a CD4 + T cell deficiency with 109 CD4 + T cells/µl (normal values: 300–1400/µL) of whole blood.

At admission on March 25, 2020, a CT chest scan indicated mild bilateral pulmonary infiltrates, and community-acquired respiratory virus (CARV)-PCR testing showed positivity for SARS-CoV-2 (Ct values were 29.90 (pharyngeal swab) and 26.35 (sputum), as analyzed by Allplex™ 2019-nCoV Assay (Seegene Inc., Seoul, South Korea), respectively.

Lenalidomide maintenance treatment was paused. Laboratory examinations showed neutropenia, lymphocytopenia, and moderately increased C-reactive protein (CRP) and interleukin-6 (IL-6) levels (Supplementary Table I). However, viral RNA was not detectable in the peripheral blood. During hospitalization, clinical symptoms worsened, and the fever persisted with 39.2 °C. Important laboratory parameters, as summarized in Supp. Table I, showed worsening lymphocytopenia (CTC grade IV), neutropenia (CTC grade I-III), a temporary decrease in the number of monocytes that persisted during hospitalization, and increased IL-6 levels with a maximum on day + 6 (26.5 pg/ ml), accompanied by a slight increase in CRP, LDH, and d-dimers. No alterations in pro-calcitonin, NT-proBNP, serum-creatinine, or liver enzymes were detected. The clinical symptoms improved, and the temperature normalized from day + 8, so that the patient could be discharged on April 2 (day + 9 from COVID-19 diagnosis, and day + 12 from first symptoms). The first negative PCR result from the nasopharyngeal swab was obtained on April 2, 2020.

The patient has been seen regularly in the outpatient department since he was discharged from the hospital. Neutrophil counts regenerated to CTC grade I neutropenia by April 24 (day + 29 since COVID-19 diagnosis), so we decided to resume lenalidomide maintenance treatment.

**Fig. 1:**
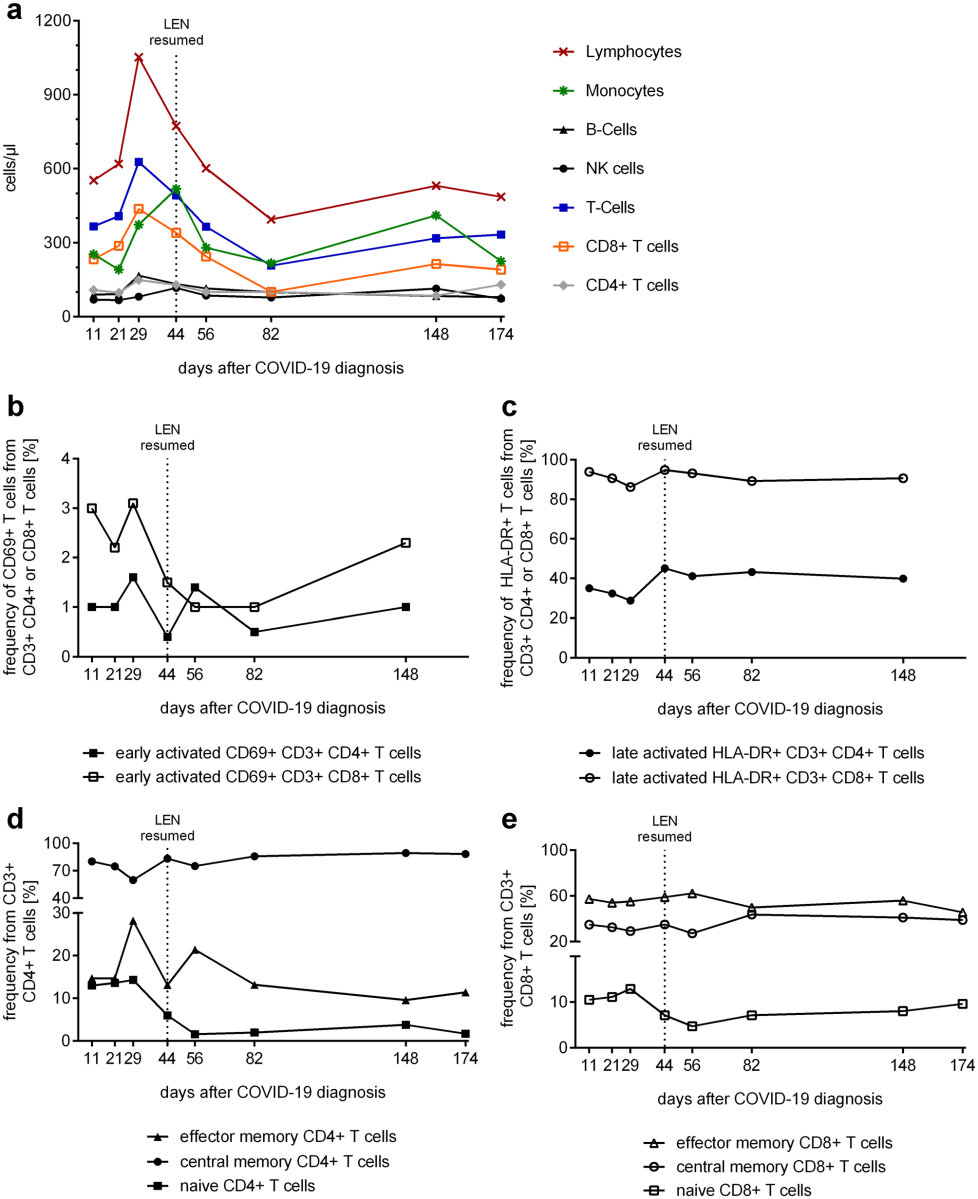
Temporal assessment of Immune cell populations, T-cell activation and T-cell subpopulations following COVID-19 diagnosis. **a** Number of immune cell populations/μL whole blood. **b** Frequency of early activated CD4 + and CD8 + T cells defined by CD69-expression. **c** Frequency of late activated CD4+ and CD8+ T cells defined by HLA-DR-expression. **d**, **e** Frequencies of naïve, effector memory, central memory CD4 + (**d**) and CD8 + (**e**) T cells

Summarized, we observed that COVID-19 took a rather mild clinical course despite pulmonary affection in an immunocompromised patient with hematologic disease. We therefore raised the question on the immunological response that had combatted COVID-19, and we continued monitoring the immune response under re-treatment with lenalidomide. We quantified innate and adaptive immune cell subpopulations by multicolor flow cytometry, specific T-cell responses to SARS-CoV-2, cytokine serum levels, and specific virus-neutralizing antibodies to SARS-CoV-2 during the course of disease and after re-introduction of lenalidomide until day + 356. Despite overall lymphocytopenia with decreased CD3+T cell numbers at COVID-19 diagnosis (Fig. 1a, Supplementary Table I), we observed a robust increase in cytotoxic CD3+ CD8+T cells with counts ranging from 233 to 438 cells/µl of whole blood. The increased CD8 + T cell numbers were accompanied by an extraordinarily strong expression of HLA-DR on 86–95% of total CD8+T-cell population, which is a marker for late activation (Fig. 1c), while CD69 expression as a marker of early activation remained constant (Fig. 1b).

In contrast, the CD3+ CD4+T helper cell population was significantly decreased with absolute values ranging from 74 cells/µl at the time of COVID-19 diagnosis to 149 cells/µl at day + 29 (Supplementary Table I). We also observed a late activation profile of these cells with up to 45% of the CD4+ T cells showing expression of HLA-DR (Fig. 1c). In regard to subsets, we only noticed a relative increase in central memory CD4 + T cells (Supplementary Table I, Fig. 1d, e). The effector memory CD4 + T population was diminished during the entire period of our immune monitoring. Interestingly, an elevated proportion of naïve CD4 + T cells and diminished proportion of CD4 + memory cells were identified as predictive markers for the severity of COVID-19, whereas a shortage in total CD4 + T cell counts was also identified per se as an unfavorable laboratory finding in another study [8].

CD19 + B-cell and CD3-/CD56+ NK-cell levels were diminished at COVID-19 diagnosis, regenerated during the observation period, but started to decrease after lenalidomide re-introduction (Supplementary Table I).

**Fig. 2:**
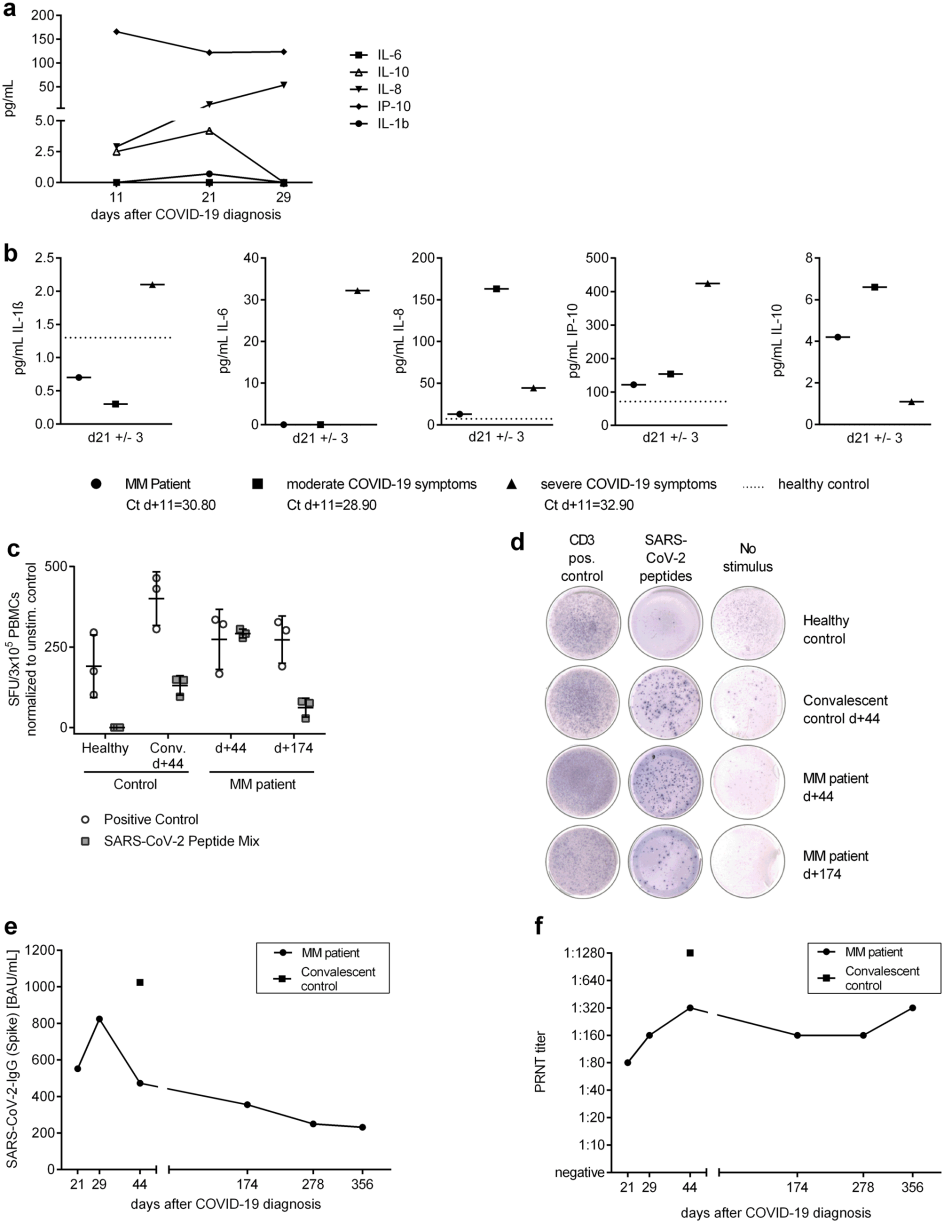
Profile of predominant cytokines, memory T cell and humoral response. **a** Development of detectable cytokines in serum of the multiple myeloma (MM) patient over time. **b** Comparison of detected cytokine concentrations between SARS-CoV-2-infected MM patient and male COVID-19 patients with severe (*n* = 1, 52 years old) and moderate (*n* = 1, 65 years old) symptoms at day 21 ± 3 days after diagnosis of SARS-CoV-2 infection. The dotted line indicates the values measured in a male healthy control (*n* = 1, < 50 years old). Ct-values of SARS-CoV-2-specific PCR at day + 11 after COVID-19 diagnosis are indicated underneath the respective patients. At day + 22, all patients were found to be negative for SARS-CoV-2. **c**, **d** IFN-γ response of cryopreserved, thawed, and overnight rested PBMCs to SARS-CoV-2 peptide mix measured by ELISpot assay. Comparison of PBMCs from MM patient isolated at day + 44 and + 174 with an age-matched, male convalescent patient (*n* = 1, 52 years old) at day + 44 after COVID-19 diagnosis and the same healthy male (*n* = 1, < 50 years) used for the other analyses. Quantification of spot forming units (SFU)/3 × 105 PBMCs was normalized to the unspecific response (SFU/3 × 105 PBMCs without stimulus) (**c**) and representative images of the wells (**d**) are displayed. Values are indicated with mean and standard derivation. **e** Quantitative detection of SARS-CoV-2 Spike protein IgG in the MM patient’s serum to different time points after COVID-19 diagnosis in comparison to an age-matched male convalescent patient (*n* = 1, 52 years old, mild COVID-19 symptoms, day + 44 after diagnosis). **f** Maximal dilution factor of serum by which SARS-CoV-2 neutralization was mediated in a plaque reduction neutralization test (PRNT). Comparison of the MM patient’s serum to different time points after COVID-19 diagnosis to an age-matched male convalescent patient (*n* = 1, 52 years old, mild COVID-19 symptoms, day + 44 after diagnosis)

Moreover, we followed up on innate immunity-derived cytokine response by monitoring Interleukin-1β (IL-1β), IL-6, Interleukin-8 (IL-8), Interleukin-10 (IL-10), and IP-10 (CXCL10, marker for interferon-γ) levels (Fig. 2a). To compare and rank the measured cytokine values into the COVID-19 landscape, we co-evaluated cytokine profiles from an age-matched patient with severe (= WHO scale 6) and with moderate COVID-19 symptoms (< WHO scale 4: Fig. 2b). As indicated by moderately elevated IP-10, the innate immune response was reflected by moderate induction of interferon-γ (IFN-γ, Fig. 2b) [9]. Although our patient had significantly elevated IL-6 levels during the hospitalization period, the levels normalized as the symptoms improved (Supplementary Table I, Fig. 2a). This was unlike to a critically ill control patient, whose IL-6, IL-1β, and IP-10 levels persisted at high levels even 3 weeks after the COVID-19 diagnosis. In contrast, IL-10, known as anti-inflammatory regulator of immunity to infection [10], was strongly elevated in the myeloma patient but low in a critically ill COVID-19 patient.

Next, we performed an IFN-γ ELISpot assay with patient’s PBMCs that were collected at day + 44 and day + 174, to examine the specific effector memory T-cell response to SARS-CoV-2 (Fig. 2c, d). Despite low initial naïve T-cell counts, the patient was able to develop specific T-cell memory subsets that showed a type II interferon reactivity to peptides from SARS-CoV-2 membrane, nucleocapsid, and spike protein to the time points tested. Additionally, this specific T-cell-dependent immunity appeared to be long lasting as reactive T cells persisted at day + 174. We additionally confirmed the presence of SARS-CoV-2-specific IFN-γ producing cells in two other MM patients to early (day + 16/ day + 22) and later time points (day + 44/ + 127) after COVID-19 diagnosis, underlining that MM patients develop a cellular memory response upon SARS-CoV-2 infection (Supplementary Fig. 1).

The next finding was the detection of anti-SARS-CoV-2 Spike IgM and anti-SARS-CoV-2 Nucleocapsid IgG antibodies in the patient’s serum at early timepoints after infection (Supplementary Table I). Remarkably, quantitative anti-SARS-CoV-2 type IgG antibody detection was in parallel conducted and showed the presence of anti-SARS-CoV-2 spike IgG antibodies (Fig. 2e) and neutralizing antibodies (Fig. 2f) until day + 356. Similar results were obtained for two further MM patients for a time period of up to day + 127 (Supplementary Fig. 1).

In the case described here, the strong immune response is notable regarding pre-existing deficiency of CD4 + T cells and reduced IgA und IgM levels due to induction chemotherapy long before SARS-CoV-2 diagnosis. It reflects that not the absolute CD4 + T cells values, but their capacity to get activated and to differentiate into memory CD4 + T cells, could be crucial for completion of effective immunological response to SARS-CoV-2.

## Conclusion

We summarize that the immune response seen in this immunocompromised patient was modest, but specific and sufficient for virus eradication. After re-introduction of lenalidomide maintenance treatment, specific IgG antibody levels, their virus-neutralizing capacities, as well as anti-SARS-CoV-2 reactive T-cells remained detectable in a 1-year view, indicating persisting immunity despite the known immune modifying effects of this drug. Hematologic malignancies per se might not be the crucial factor that is affecting the course of COVID-19.

## Supplementary Information

Below is the link to the electronic supplementary material.

Supplementary file1 (DOCX 447 KB)

## Data Availability

The data and material will be made available upon request.

## References

[1] Zhu N, Zhang D, Wang W, Li X, Yang B, Song J, Zhao X, Huang B, Shi W, Lu R et al (2020) A novel coronavirus from patients with pneumonia in China N Engl J Med 382:727–733. 10.1056/NEJMoa2001017. Epub 2020 Jan 24. PMID: 31978945; PMCID: PMC7092803 10.1056/NEJMoa2001017 PMC7092803 31978945

[2] Hultcrantz M, Richter J, Rosenbaum C, Patel D, Smith E, Korde N, Lu S, Mailankody S, Shah U, Lesokhin A et al (2020) COVID-19 infections and outcomes in patients with multiple myeloma in New York City: a cohort study from five academic centers. 10.1158/2643-3230.BCD-20-0102. PMID: 32577667; PMCID: PMC7302217 10.1158/2643-3230.BCD-20-0160 PMC8510790 34661147

[3] Engelhardt M, Shoumariyeh K, Rösner A, Ihorst G, Biavasco F, Meckel K, von Metzler I, Treurich S, Hebart H, Grube M et al (2020) Clinical characteristics and outcome of multiple myeloma patients with concomitant COVID-19 at Comprehensive Cancer Centers in Germany. Haematologica 105:2872–2878. 10.3324/haematol.2020.262758. Epub ahead of print. PMID: 32732357 10.3324/haematol.2020.262758 PMC7716370 33256391

[4] Terpos E, Engelhardt M, Cook G, Gay F, Mateos MV, Ntanasis-Stathopoulos I, van de Donk NWCJ, Avet-Loiseau H, Hajek R, Vangsted AJ (2020). Management of patients with multiple myeloma in the era of COVID-19 pandemic: a consensus paper from the European Myeloma Network (EMN). Leukemia.

[5] Kohmer N, Westhaus S, Rühl C, Ciesek S, Rabenau HF (2020). Brief clinical evaluation of six high-throughput SARS-CoV-2 IgG antibody assays. J Clin Virol.

[6] Kohmer N, Rühl C, Ciesek S, Rabenau HF (2021). Utility of different surrogate enzyme-linked immunosorbent assays (sELISAs) for detection of SARS-CoV-2 neutralizing antibodies. J Clin Med.

[7] Huang C, Wang Y, Li X, Ren L, Zhao J, Hu Y, Zhang L, Fan G, Xu J, Gu X (2020). Clinical features of patients infected with 2019 novel coronavirus in Wuhan, China. Lancet.

[8] Qin C, Zhou L, Hu Z, Zhang S, Yang S, Tao Y, Xie C, Ma K, Shang K, Wang W et al (2020) Dysregulation of immune response in patients with COVID-19 in Wuhan, China. Clin Infect Dis 71:762–768. 10.1093/cid/ciaa248 10.1093/cid/ciaa248 PMC7108125 32161940

[9] Rotondi M, Lazzeri E, Romagnani P, Serio M (2003). Role for interferon-gamma inducible chemokines in endocrine autoimmunity: an expanding field. J Endocrinol Invest.

[10] Couper KN, Blount DG, Riley EM (2008). IL-10: the master regulator of immunity to infection. J Immunol.

