# Supplementary material for: COVID-19 in multiple-myeloma patients: cellular and humoral immunity against SARS-CoV-2 in a short- and long-term view
Source: J Mol Med (Berl). 2021 Oct 18;100(3):463–70. doi: 10.1007/s00109-021-02114-x (PMC8520766; doi:10.1007/s00109-021-02114-x)
Supplement: Supplementary file 1 — Supplementary file1 (DOCX 447 KB) [file 109_2021_2114_MOESM1_ESM.docx]

**Supplementary Table I: Laboratory parameters at different time points after COVID-19 diagnosis**.

| **Laboratory parameter** | **Range/Unit** | **d+1** | **d+6** | **d+11** | **d+21** | **d+29** | **d+44** | **d+56** | **d+82** | **d+148** | **d+174** | **d+278** | **d+356** |
| --- | --- | --- | --- | --- | --- | --- | --- | --- | --- | --- | --- | --- | --- |
|  |  | **Lenalidomid withdrawn** | | | | | **Lenalidomid resumed** | | | | | | |
| IL-6 | < 7 pg/ml | **12.6 +** | **26.5 +** | 1.6 | 1.7 | n.d. | 3.8 | n.d. | n.d. | n.d. | n.d. | n.d. | n.d. |
| CRP | < 0.5 mg/dl | **2.8 +** | **6.96 +** | **0.17 +** | 0.05 | 0.06 | 0.1 | 0.05 | 0.08 | 0.67 | 0.07 | n.d. | n.d. |
| D-Dim | < 500 ng/ml | 420 | **569 +** | 326 | n.d. | 346 | n.d. | n.d. | n.d. | n.d. | n.d. | n.d. | n.d. |
| LDH | < 248 U/l | 214 | **278 +** | 200 | 166 | 195 | 179 | 180 | n.d. | n.d. | n.d. | n.d. | n.d. |
| WBC | 3.92-9.81/nl | **2.05 -** | **2.67 -** | **3.05 -** | **1.96 -** | **3.36 -** | 4.04 | **2.2 -** | **2.01** | 4.83 | **2.42** | n.d. | **2.43-** |
| **Neutrophils** | 1.78-6.23/nl | **1.16 -** | 1.8 | 2.02* | **0.98 -** | **1.6 -** | 2.53 | **1.08 -** | **1.24** | 3.37 | **1.09** | n.d. | **1.37-** |
| **Monocytes** | 0.26-0.87/nl | 0.28 | **0.16 -** | 0.33 | 0.26 | 0.48 | 0.53 | 0.36 | 0.27 | 0.47 | 0.28 | n.d. | 0.32 |
| **Lymphocytes** | 1.05-3.24/nl | **0.57 -** | **0.12 -** | **0.56 -** | **0.62 -** | 1.052 | **0.77 -** | **0.54 -** | **0.37-** | **0.52-** | **0.51-** | n.d. | **0.51-** |
| CD3+ | 700-2100/µl | **422 -** | n.d. | **366 -** | **408 -** | **627 -** | **492 -** | **365 -** | **207 -** | **318 -** | **333 -** | n.d. | **293-** |
| CD3+CD4+ | 300-1400/µl | **74 -** | n.d. | **108 -** | **97 -** | **149 -** | **129 -** | **101 -** | **100 -** | **85 -** | **130 --** | n.d. | **125-** |
| CD3+CD8+ | 200-900/µl | 324 | n.d. | 233 | 287 | 438 | 340 | 244 | **101** - | 214 | **119 -** | n.d. | **158-** |
| B cells CD19+ | 100-500/µl | n.d. | n.d. | **89 -** | **92 -** | 167 | 133 | 115 | **99** - | **84** - | **79 -** | n.d. | **74-** |
| NK cells  CD56+CD3- | 90-600\|µl | n.d. | n.d. | **69 -** | **67 -** | **81 -** | 117 | **86 -** | **78 -** | 114 | **73 -** | n.d. | 128 |
|  | **CD4+ T helper cell subsets** | | | | | | | | | | | | |
| Naive CD4+ *CD4+CD45RA+CD62L+* | 121-456/µl | n.d. | n.d. | **14 --** | **13 --** | **21 --** | **8 --** | **2 --** | **2 --** | **3 --** | **2 --** | n.d. | n.d. |
| Effector memory CD4+ *CD4+CD45RO+CD62L-* | 59-321/µl | n.d. | n.d. | **16 -** | **14 -** | **42 -** | **17 -** | **22 -** | **13 -** | **8 -** | **15 -** | n.d. | n.d. |
| Central memory CD4+ *CD4+CD45RO+CD62L+* | 92-341/µl | n.d. | n.d. | **86 -** | **72 -** | **89 -** | 107 | **76 -** | **86 -** | **76 -** | 115 | n.d. | n.d. |
| T regs *CD4+CD25bright +CD127dim* | 25-180/µl | n.d. | n.d. | **17 -** | **10 -** | **15 -** | **19 -** | **15 -** | **17 -** | **14 -** | **17 -** | n.d. | n.d. |
|  | **CD8+ cytotoxic T cell subsets** | | | | | | | | | | | | |
| Naive CD8+ *CD8+CD45RA+CD62L+* | 86-257/µl | n.d. | n.d. | **24 -** | **32 -** | **57 -** | **24 -** | **11 -** | **7 -** | **17 -** | **18 -** | n.d. | n.d. |
| Effector memory CD8+ *CD8+CD45RO+CD62L-* | 15-162/µl | n.d. | n.d. | 133 | 155 | **241 +** | **200 +** | 152 | 50 | 119 | 87 | n.d. | n.d. |
| Central memory CD8+ *CD8+CD45RO+CD62L+* | 19-93/µl | n.d. | n.d. | 81 | 93 | **128 +** | **119 +** | 66 | 44 | 88 | 74 | n.d. | n.d. |
|  | **T cell activation** | | | | | | | | | | | | |
| Early activated CD4+ *CD4+CD69+* | 0-70/µl | n.d. | n.d. | 1 | 1 | 2 | 1 | 1 | 1 | 1 | n.d. | n.d. | n.d. |
| Early activated CD8+ *CD8+CD69+* | 0-90/µl | n.d. | n.d. | 7 | 6 | 14 | 5 | 2 | 1 | 5 | n.d. | n.d. | n.d. |
| Late activated CD4+ *CD4+HLA-DR+* | 0-70/µl | n.d. | n.d. | 38 | 31 | 43 | 58 | 42 | 43 | 34 | n.d. | n.d. | n.d. |
| Late activated CD8+ *CD8+HLA-DR+* | 0-90/µl | n.d. | n.d. | **219 +** | **260 +** | **378 +** | **322 +** | **227 +** | 90 | **194 +** | n.d. | n.d. | n.d. |
|  | **Humoral response** | | | | | | | | | | |  |  |
| Anti-Spike IgM | quali-tative | n.d. | n.d. | n.d. | **Pos.** | **Pos.** | **Pos.** | n.d. | n.d. | n.d. | Neg. | Neg. | Neg. |
| Anti-Nucleocapsid IgG | quali-tative | n.d. | n.d. | n.d. | **Pos.** | **Pos.** | **Pos.** | n.d. | n.d. | n.d. | **Pos.** | Neg. | Neg. |
| Anti-Spike IgG quantitative | BAU/ml | n.d. | n.d. | n.d. | **551.4** | **823.5** | **472.6** | n.d. | n.d. | n.d. | **355.1** | **249.6** | **231.2** |
|  | Interp.. | n.d. | n.d. | n.d. | **Pos.** | **Pos.** | **Pos.** | n.d. | n.d. | n.d. | **Pos.** | **Pos.** | **Pos.** |
| Verification SARS-CoV-2 IgG | quali-tative | n.d. | n.d. | n.d. | **Pos.** | **Pos.** | **Pos.** | n.d. | n.d. | n.d. | **Pos.** | **Pos.** | **Pos.** |
| PRNT | Titer | n.d. | n.d. | n.d. | **1:80** | **1:160** | **1:320** | n.d. | n.d. | n.d. | **1:160** | **1:160** | **1:320** |
|  | Interp. | n.d. | n.d. | n.d. | **Pos.** | **Pos.** | **Pos.** | n.d. | n.d. | n.d. | **Pos.** | **Pos.** | **Pos.** |

**after G-CSF as on day +8 the neutrophils were 0.5/µl; LEN = lenalidomide; n.d. = not done; Pos. = positive; Neg. = negative; Interp.= interpretation; BAU = binding antibody units; PRNT = plaque reduction neutralization test*

**Supplementary Table II: Humoral response of Convalescent patient and two further MM patients after COVID-19 diagnosis**.

| **Laboratory parameter** | **Unit** | **d+16** | **d+22** | **d+127** | **d+22** | **d+44** |
| --- | --- | --- | --- | --- | --- | --- |
|  | **Patient characteristic** | | | | | |
| Patient ID |  | MM Patient 2 | | | MM Patient 3 | Convalescent patient |
| Age | years | 74 | | | 59 | 52 |
| Sex | male/female | female | | | female | male |
| MM type/ therapy |  | IgA lambda, no current therapy | | | IgG lambda, on Lenalidomide treatment |  |
|  | **Humoral response** | | | | | |
| Anti-Spike IgM | qualitative | **Pos.** | **Pos.** | **Neg.** | **Pos.** | **Pos.** |
| Anti-Nucleocapsid IgG | qualitative | **Pos.** | **Pos.** | **Pos.** | **Pos.** | **Pos.** |
| Anti-Spike IgG2 quantitative | BAU/mL | **290.3** | **660.7** | **306.1** | **3543.7** | **1023.2** |
|  | Interpretation | **Pos.** | **Pos.** | **Pos.** | **Pos.** | **Pos.** |
| Verification SARS-CoV-2 IgG | qualitative | **Pos.** | **Pos.** | **Pos.** | **Pos.** | **Pos.** |
| PRNT | Titer | **1:40** | **1:160** | **1:80** | **1:160** | **>1:1280** |
|  | Interpretation | **Pos.** | **Pos.** | **Pos.** | **Pos.** | **Pos.** |

*Pos. = positive; Neg. = negative; BAU = binding antibody units; PRNT = plaque reduction neutralization test*

**

**

**Supplementary Figure 1**

Profile of SARS-CoV-2 specific memory T cell response in two further multiple myeloma patients

(a-b) IFN-γ response of cryopreserved, thawed and overnight rested PBMCs to SARS-CoV-2 peptide mix measured by ELISpot assay. Comparison of PBMCs from MM Patient 2 isolated at day +16 and +127, and from Patient 3 isolated at day +22 and +44 and the same healthy male (n=1, <50 years) used for the other analyses. Quantification of spot forming units (SFU)/3x105 PBMCs was measured in duplicates or triplicates and normalized to the unspecific response (SFU/3x105 PBMCs without stimulus). Displayed are the single measured values ± standard derivation.
